# Denning in brown bears

**DOI:** 10.1002/ece3.6372

**Published:** 2020-05-25

**Authors:** Enrique González‐Bernardo, Luca Francesco Russo, Esther Valderrábano, Ángel Fernández, Vincenzo Penteriani

**Affiliations:** ^1^ Research Unit of Biodiversity (UMIB, CSIC‐UO‐PA) Mieres Spain; ^2^ Pyrenean Institute of Ecology (IPE‐CSIC) Zaragoza Spain; ^3^ Department of Biosciences and the Territory Università degli Studi del Molise Pesche Italy; ^4^ COPAR Research Group Faculty of Veterinary University of Santiago de Compostela Lugo Spain; ^5^ Biosfera Consultoría Medioambiental Oviedo Spain

**Keywords:** climate change, den chronology, den selection, hibernation, physiology, *Ursus arctos*

## Abstract

1. Hibernation represents an adaptation for coping with unfavorable environmental conditions. For brown bears *Ursus arctos*, hibernation is a critical period as pronounced temporal reductions in several physiological functions occur.
2. Here, we review the three main aspects of brown bear denning: (1) den chronology, (2) den characteristics, and (3) hibernation physiology in order to identify (a) proximate and ultimate factors of hibernation as well as (b) research gaps and conservation priorities.
3. Den chronology, which varies by sex and reproductive status, depends on environmental factors, such as snow, temperature, food availability, and den altitude. Significant variation in hibernation across latitudes occurs for both den entry and exit.
4. The choice of a den and its surroundings may affect individual fitness, for example, loss of offspring and excessive energy consumption. Den selection is the result of broad‐ and fine‐scale habitat selection, mainly linked to den insulation, remoteness, and availability of food in the surroundings of the den location.
5. Hibernation is a metabolic challenge for the brown bears, in which a series of physiological adaptations in tissues and organs enable survival under nutritional deprivation, maintain high levels of lipids, preserve muscle, and bone and prevent cardiovascular pathologies such as atherosclerosis.

It is important to understand: (a) proximate and ultimate factors in denning behavior and the difference between actual drivers of hibernation (i.e., factors to which bears directly respond) and their correlates; (b) how changes in climatic factors might affect the ability of bears to face global climate change and the human‐mediated changes in food availability; (c) hyperphagia (period in which brown bears accumulate fat reserves), predenning and denning periods, including for those populations in which bears do not hibernate every year; and (d) how to approach the study of bear denning merging insights from different perspectives, that is, physiology, ecology, and behavior.

## Graphical abstract

The period of hibernation in the brown bear allows it to face the adverse climatic conditions of the scarce of food during the winter. In this study, we review the published literature on this topic and approach it from the perspective of den chronology, den selection, and physiology of hibernation. We elucidate the importance of understanding (a) the proximate and ultimate factors in denning behavior and the difference between actual drivers of hibernation and their correlates; (b) how changes in climatic factors might affect the ability of bears to face global climate change; (c) the predenning stage; (d) hyperphagia, predenning and denning periods; and (e) how to approach the study of bear denning merging insights from different perspectives.

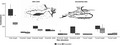

## 1 INTRODUCTION

Hibernation is an important life history activity that coincides with winter in seasonal environments and represents an adaptation for coping with harsh environmental conditions, generally associated with low temperatures and low food abundance (Geiser, 2013; Ruf & Geiser, 2015).

For brown bears *Ursus arctos*, hibernation is a critical period because at that time (Friebe, Swenson, & Sandegren, 2001; Geiser, 2004; Haroldson, Ternent, Gunther, & Schwartz, 2002; Linnell, Swenson, Andersen, & Barnes, 2000): (a) pregnant females give birth and undergo lactation while in dens; (b) energy savings during hibernation can be substantial; and (c) premature exit can negatively affect energy conservation and cub survival (Pigeon, Stenhouse, & Côté, 2016). Thus, the conservation and management of brown bears requires knowledge regarding the denning ecology of different populations. Moreover, hibernation demands a preceding phase (hyperphagia) involving the intense search for food in order to store energy, and bears may spend as much as half of their life in winter dens (Friebe et al., 2001). Prior to hibernation, brown bears select specific denning sites as well as dens and, while in dens, bears show pronounced temporal reductions in several physiological functions and do not feed or drink (Hellgren, 1998; Linnell et al., 2000). Finally, the choice of the brown bear as an interesting case study is based on two additional reasons: (a) the existence of new research accumulated in the recent years on the hibernation of the species; and (b) for being a species widely distributed around the northern hemisphere, with populations of different characteristics inhabiting very diverse habitats. This review thus allows identifying patterns or gradients of denning behavior throughout the distribution range of the species and highlighting possible differences between populations.

Here, we review the three main aspects of brown bear denning, that is, (1) den chronology, (2) den characteristics, and (3) hibernation physiology to provide an up‐to‐date assessment of this crucial phase of brown bear biology and to identify research gaps and conservation priorities during this life stage. When possible, we aimed to highlight commonalities and differences both within and among different bear populations and the underlying mechanisms. In particular, we expect that: (a) den chronology may vary by sex and bear reproductive status; (b) the duration of hibernation also depends on environmental factors, that is, snow, temperature, and food availability; (c) a relationship may exist between denning period and latitude, longitude and altitude; and (d) although the choice of a den and den surroundings may be variable, some differences may still exist by class, age, and sex. Taking the opportunity to review a topic with a vast scientific literature, we also aimed to identify ultimate (i.e., factors which in the course of evolution have shaped, through natural selection, biological processes, and behaviors) and proximate (i.e., external stimuli which initiate or maintain biological processes and behaviors) factors in denning behavior, particularly regarding the behavior of individuals of different reproductive categories, ages, sexes, and populations. This approach will also allow for clarifying actual drivers, that is, factors to which bears directly respond, and their correlates, that is, factors that may be correlated to actual drivers.

## 2 METHODS

The search for articles related to the theme of brown bear denning behavior and hibernation was carried out until March 2020 using Google Scholar and Thomson Reuters “Web of Science” (Scopus) databases. We conducted a literature review using a broad range of terms that represent the variety of ways in which bear denning behavior and hibernation may be included. The terms “bear,” “grizzly,” and “*Ursus arctos*” were combined with the following terms: “den,” “denning chronology,” “denning ecology,” “den entry,” “den exit,” “hibernation,” “hibernation driver,” and “phenology.” We also searched in the literature‐cited section of all retrieved articles. We primarily selected studies conducted on free‐ranging brown bears, which were organized according to the three main themes concerning hibernation: den chronology (*n* = 45 papers), den characteristics and surroundings (*n* = 42 papers), and physiology of hibernation (*n* = 61 papers). Finally, we added necessary references to complete the introduction and discuss the results (e.g., other hibernating mammals and Global Change). Possible variations in hibernation period across latitudes and longitudes for both den entry and exit where tested by Pearson's correlations, while a Spearman correlation was used to test the relationship between den entry and exit with altitude.

## 3 RESULTS

### 3.1 Den chronology

#### 3.1.1 Predenning period

After hyperphagia, during which bears accumulate the energy necessary for hibernation, individuals gradually decrease their rhythms of activity and movements before den entry (Friebe et al., 2001; Manchi & Swenson, 2005; Sahlén, Støen, & Swenson, 2011). This phase, called predenning, which generally lasts between one and two weeks (Friebe et al., 2001; Manchi & Swenson, 2005; Sahlén et al., 2011), may also go on for more than one month (Evans et al., 2016; Servheen & Klaver, 1983). Alongside the decrease in activity, physiological changes occur during the predenning period (Evans et al., 2016).

This period may vary among the different categories and age classes of bears (Manchi & Swenson, 2005; Sahlén, Ordiz, Swenson, & Støen, 2015). For example, adult males and females with offspring generally arrive in denning sites after pregnant females, solitary females, and females with cubs‐of‐the‐year (Sahlén et al., 2011). Probably due to their greater experience and knowledge of their home range (Manchi & Swenson, 2005), older bears seem to spend less time than younger individuals in the denning area before hibernation (Sahlén et al., 2011; Sahlén, Friebe, Sæbø, Swenson, & Støen, 2015). Finally, the length of the predenning period can also vary across years (Manchi & Swenson, 2005; Sahlén, Friebe, et al., 2015).

#### 3.1.2 Influence of sex and reproductive status

**FIGURE 1:**
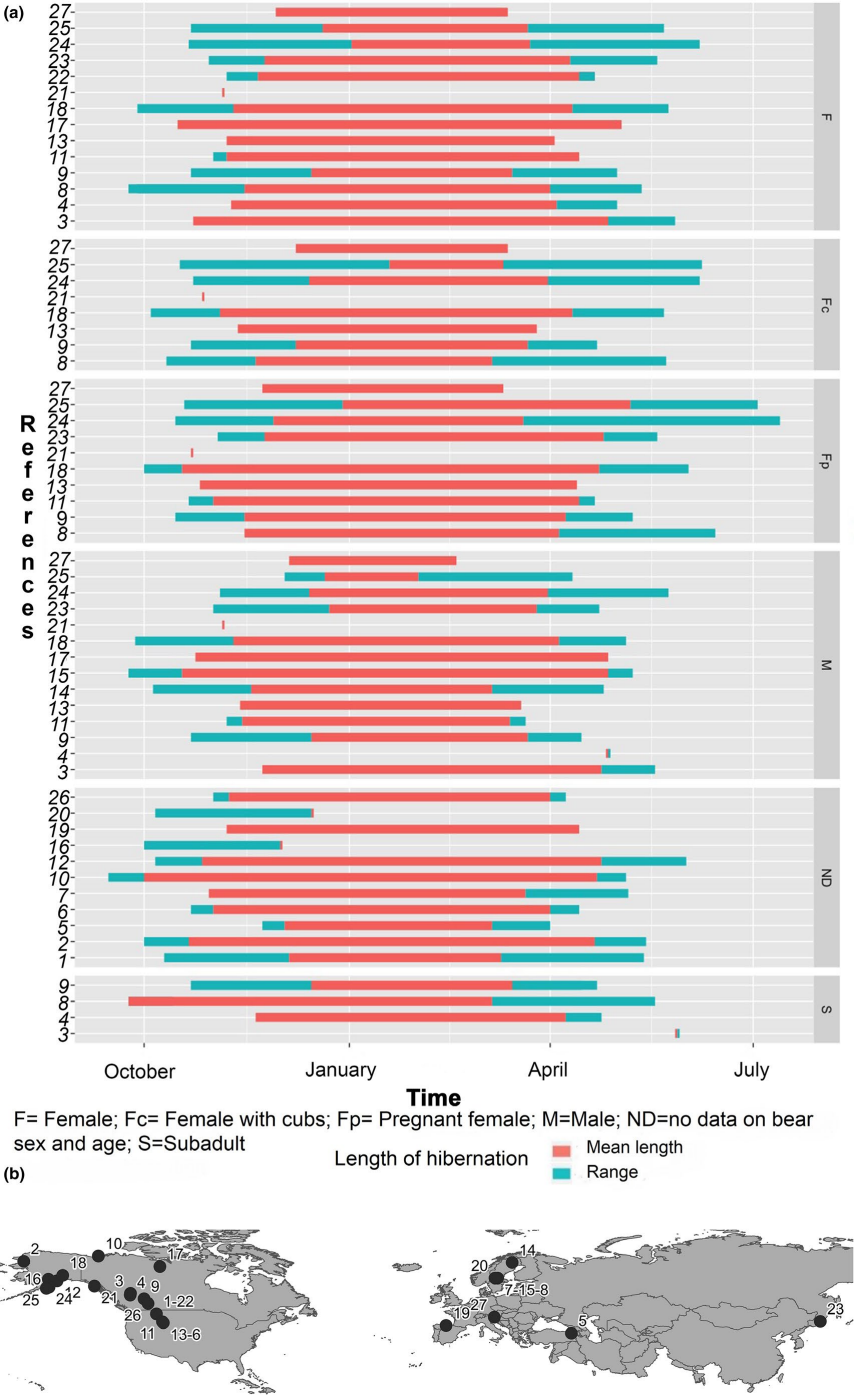
(a) Duration of brown bear hibernation by age class: F = female, Fc = female with cubs, Fp = pregnant female, M = male, S = Subadult, and ND = no data available. Red lines represent the average duration of hibernation; cyan lines represent the first date of den entry and the last date of den exit. (B) Geographical locations of the areas in which the cited studies on the duration of brown bear hibernation have been carried out. References for the Figure 1: 1. Aune (1994); 2. Ballard, Ayres, Roney, Reed, and Fancy (1991); 3–4. Ciarniello et al. (2005); 5. Cozzi et al. (2016); 6. Craighead & Craighead, 1972; 7. Evans et al. (2016); 8. Friebe et al. (2001); 9. Graham and Stenhouse (2014); 10. Harding (1976); 11. Haroldson et al. (2002); 12. Hilderbrand et al. (2000); 13. Judd et al. (1986); 14–15. Manchi and Swenson (2005); 16. Mangipane et al. (2018); 17. McLoughlin, Case, et al. (2002), McLoughlin, Cluff, et al. (2002); 18. Miller (1990); 19. Naves and Palomero (1993); 20. Sahlén, Friebe, et al. (2015); 21. Schoen et al. (1987); 22. Servheen and Klaver (1983); 23. Seryodkin et al. (2003); 24–25. Van Daele et al. (1990); 26. Vroom et al. (1980); 27. Krofel et al. (2017)

Similarly, den chronology also varies by sex and reproductive status (Figure 1), with females entering the den earlier and leaving later compared with males (Ciarniello, Boyce, Heard, & Seip, 2005; Craighead & Craighead, 1972; Haroldson et al., 2002; Judd, Knight, & Blanchard, 1986; Manchi & Swenson, 2005; McLoughlin, Case, et al., 2002; McLoughlin, Cluff, & Messier, 2002; Pigeon, Stenhouse, et al., 2016; Schoen, Beier, Lentfer, & Johnson, 1987; Van Daele, Barnes, & Smith, 1990). Specifically, pregnant females enter the den earlier and exit later than other bear classes, the latter probably because of their need to spend more time inside the den to take care of new‐born cubs, as well as the limited mobility of the cubs in the first weeks of life (Ciarniello et al., 2005; Friebe et al., 2001; Graham & Stenhouse, 2014; Haroldson et al., 2002; Judd et al., 1986; Manchi & Swenson, 2005; McLoughlin, Case, et al., 2002; McLoughlin, Cluff, et al., 2002; Pigeon, Stenhouse, et al., 2016; Planella et al., 2019; Schoen et al., 1987; Van Daele et al., 1990). Generally, the bear groups that enter hibernation dens few time after pregnant females are females with cubs and lone adult females (Friebe et al., 2001; Pigeon, Stenhouse, et al., 2016; Van Daele et al., 1990). Finally, males and subadults seem to generally have shorter denning periods than adult females (i.e., parturient females, females with yearlings, or solitary females; Ciarniello et al., 2005; Haroldson et al., 2002; Judd et al., 1986; Krofel, Špacapan, & Jerina, 2017; Manchi & Swenson, 2005; McLoughlin, Case, et al., 2002; McLoughlin, Cluff, et al., 2002; Pigeon, Stenhouse, et al., 2016; Schoen et al., 1987; Van Daele et al., 1990).

The earlier arrival at denning sites and longer hibernation of pregnant females compared to other bear classes may occur because the denning chronology of pregnant females is mainly determined by an ultimate reason, namely reproductive needs, whereas other bear classes are mostly governed by a trade‐off between proximate (environmental conditions) and ultimate (metabolic dietary‐related needs, energy conservation) factors.

#### 3.1.3 Influence of environmental factors

The duration of hibernation can also depend on (proximate) environmental factors, that is, snow, temperature, and food availability. As a general rule, the duration of hibernation in different brown bear populations seems to be conditioned by both snowfall/snow depth in autumn (Akhremenko & Sedalishchev, 2008; Craighead & Craighead, 1972; Friebe et al., 2001; Manchi & Swenson, 2005) and snowmelt in spring (Pigeon, Stenhouse, et al., 2016; Schoen et al., 1987). Snowfall can act as a major impetus to begin hibernation (Craighead & Craighead, 1972; Friebe et al., 2001; Manchi & Swenson, 2005; Reynolds, Curatolo, & Quimby, 1976; Servheen & Klaver, 1983), with bears generally entering dens after first snowfall (Craighead & Craighead, 1972; Evans et al., 2016; Manchi & Swenson, 2005), although occasional snowstorms seem not to act as a stimulus for den entry (Judd et al., 1986; Van Daele et al., 1990). However, no significant correlations were detected between den entry/exit and snow depth (Delgado et al., 2018; Judd et al., 1986). Likewise, Bojarska, Drobniak, Jakubiec, and Zyśk‐Gorczyńska (2019) reported a probability of observations of bears negatively correlated with depth of snow cover.

Another important proximate factor affecting den entry/exit is ambient temperature (Craighead & Craighead, 1972; Delgado et al., 2018; Evans et al., 2016; Friebe et al., 2014; Manchi & Swenson, 2005; McLoughlin, Case, et al., 2002; McLoughlin, Cluff, et al., 2002; Pigeon, Stenhouse, et al., 2016). In Scandinavia, Evans et al. (2016) observed that the average (mean ± *SE*) daily temperature when bears enter dens is 1.03 ± 0.95°C and that ambient temperature is associated with a decrease in body temperature which, consequently, results in a change in heart rate (Evans et al., 2016). When bears leave the den, the daily mean ambient temperature is 3.7 ± 1.3°C and their mean body temperature is 36.7 ± 0.15°C. A decrease in the length of the hibernation period and the postponement of den entry may be associated with warm winters (Evans et al., 2016), whereas low autumn temperatures may cause early den entry (Friebe et al., 2014). However, Pigeon, Stenhouse, et al. (2016) considered that autumn temperatures may have a minor role in den entry dates. Some studies reported that den emergence might be somewhat regulated by temperature increase (McLoughlin, Case, et al., 2002; McLoughlin, Cluff, et al., 2002; Manchi & Swenson, 2005; González‐Bernardo, Bombieri, Delgado, Penteriani, & Press, in press), and Evans et al. (2016) showed that den exit was not dependent on the exact ambient temperature on the day of exit, probably because den emergence is a longer process in which physiology is tightly integrated with ambient temperature. In Poland, brown bear winter activity has been positively related to ambient temperature (Bojarska et al., 2019). However, brown bear sensitivity to changes in climatic conditions varies as a function of den entry and exit dates (Delgado et al., 2018). Indeed, brown bears are most sensitive to climatic variations around first exit and last entry dates, that is, a change in ambient temperature in periods closer to the average date that bears first enter/exit their dens has a greater influence on denning dates than during other periods (Delgado et al., 2018).

The hibernation period is primarily affected by a decrease in food availability (Pigeon, Stenhouse, et al., 2016; Schoen et al., 1987; Van Daele et al., 1990), which may also be related to the amount of snow (Pigeon, Stenhouse, et al., 2016). Some authors have hypothesized that this snow‐induced lower availability of food principally guides den entry and exit, that is, the availability of food in late autumn–early winter delays den entry (Pigeon, Stenhouse, et al., 2016; Van Daele et al., 1990). Food availability seems to affect less the den entry date of pregnant females, which start hibernating when berries are still available and abundant (Friebe et al., 2001), which supports the stronger dependence of pregnant females’ hibernation on ultimate cues. Finally, because brown bears are facultative hibernators, the continuous availability of food and mild climate may prompt individuals to winter outside dens (Huber & Roth, 1997; Nores et al., 2010; Van Daele et al., 1990).

#### 3.1.4 Influence of latitude, longitude and altitude

**FIGURE 2:**
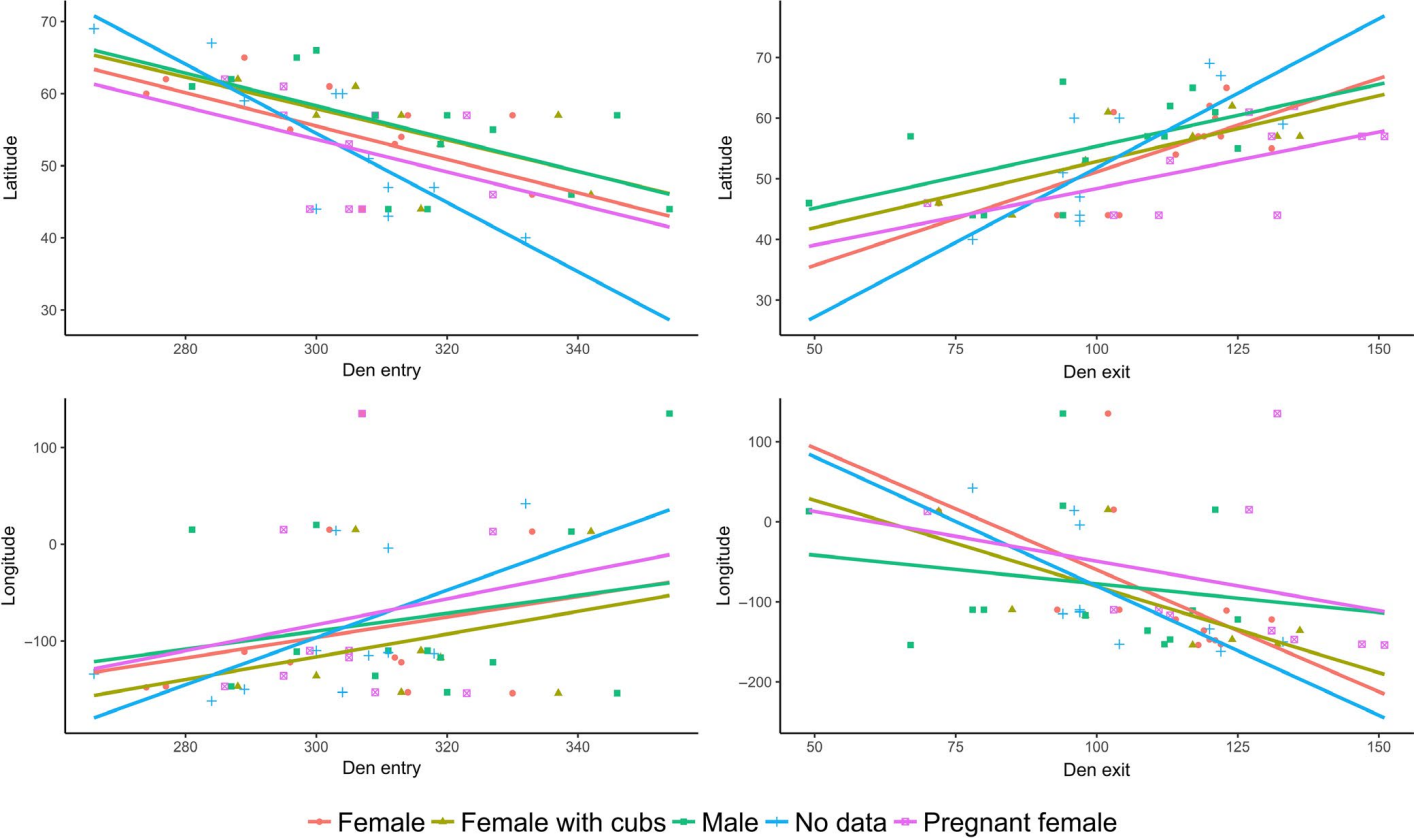
Dates of brown bear den entry and exit change according to latitude, longitude, and age class. F = female, Fc = female with cubs, Fp = pregnant female, M = male, S = Subadult, and ND = no age data available. References for the Figure 2: (Clevenger & Purroy, 1988; Craighead & Craighead, 1972; Groff et al., 1998; Huber & Roth, 1997; Judd et al., 1986; Lentfer et al., 1972; McLoughlin, Case, et al., 2002; McLoughlin, Cluff, et al., 2002; Petram et al., 2004; Pigeon, Côté, et al., 2016; Vroom et al., 1980)

As a global pattern for brown bears, individuals in southern latitudes generally enter dens later and spend less time hibernating than bears in northern latitudes (Graham & Stenhouse, 2014; Haroldson et al., 2002; Linnell et al., 2000; Manchi & Swenson, 2005; McLoughlin, Case, et al., 2002; McLoughlin, Cluff, et al., 2002; Figure 2). When comparing different studies on den chronology, we detected a significant variation in hibernation period across latitudes for both den entry (*n* = 57, *r* = −.52, *p* = .0001) and exit (*n* = 59, *r* = .48, *p* = .0001), with bears in more northern areas spending more time hibernating than bears in the southernmost latitudes (Figure 2). Proximate factors such as local weather conditions and the availability of food may be the drivers triggering the detected variations in den chronology. However, when taking into account the different classes of bears, that is, adult males, adult females, females with cubs, pregnant females, and subadults, den entry versus latitude was only significant for adult males (*n* = 12, *r* = −.58, *p* = .05) and nearly significant for adult females (*n* = 14, *r* = −.52, *p* = .06). That is, for females with cubs, pregnant females, and subadults, latitude seems to have only a minor effect on hibernation length. On the other hand, when taking into account the different classes of bears for den exit versus latitude, only adult females showed a significant correlation (*r* = .68, *p* = .008). Neither den entry (*r* = .09, *p* = .51) nor exit (*r* = −.21, *p* = .10) was correlated with longitude (Figure 2). Den altitude may also affect the duration of hibernation because of the varying climatic conditions over altitudinal gradients (Ciarniello et al., 2005; Pigeon, Stenhouse, et al., 2016), with bears denning at lower altitudes (e.g., plains, coastal areas) emerging earlier than those hibernating at higher altitudes. However, when comparing den chronology with the mean altitude of eight study areas, neither den entry (*ρ* = 0.10, *p* = .82) nor exit (*ρ* = −0.30, *p* = .47) was significantly correlated with altitude.

#### 3.1.5 Den abandonment

Den abandonment, that is, the premature leaving of a hibernation den with or without subsequent re‐denning, has been reported in different populations of brown bears. Although den abandonment occurs naturally due to flooding or winter food availability (Huber & Roth, 1997; Nores et al., 2010; Schoen et al., 1987; Van Daele et al., 1990), human activities have been reported as the main cause of den abandonment (Linnell et al., 2000; Sahlén, Friebe, et al., 2015; Swenson, Sandegren, Brunberg, & Wabakken, 1997). It has been reported how this effect is dependent on the distance at which human activity takes place (<1 km away and especially less than 200 m, den abandonment increases significantly Linnell et al., 2000). Thus, activities such as industrial and forestry activity, hunting (Sahlén, Friebe, et al., 2015; Swenson et al., 1997), transit of people (Swenson et al., 1997), or even the research activity at the surroundings of the den (Huber & Roth, 1997) have been described as causes of den abandonment. It has also been reported that abandoned dens were located at a shorter distance from roads than nonabandoned dens (Elfström & Swenson, 2009). Different studies found that if den abandonment is due to disturbances it affects equally individuals of both sexes (Krofel et al., 2017; Swenson et al., 1997), while if it is due to the presence of a food source, a male‐biased den abandonment (Krofel et al., 2017; Van Daele et al., 1990). Den abandonment can have negative consequences for populations (Linnell et al., 2000) as cub mortality increases (probability of losing at least one cub is multiplied by 10 in the case of den abandonment, Swenson et al., 1997).

Separate mention deserves the effect of the availability of food during the winter in the den abandonment. In certain populations where there are natural food sources during the winter, part of the population may not hibernate (males on Kodiak Island, Van Daele et al., 1990). This is more common in southern populations where weather conditions are less harsh and food is available permanently, such as hard mast or chestnuts (Clevenger, Purroy, & Pelton, 1992) and where it is not unusual to see bears not hibernating or interrupting hibernation (Clevenger, Purroy, & Pelton, 1990; Huber & Roth, 1997; Nores et al., 2010). However, this food‐related den abandonment is especially intense in some populations where bears have access to supplementary food during winter, which may alter the chronology of hibernation or winter activity patterns (Bojarska et al., 2019; Cozzi et al., 2016; Krofel et al., 2017).

### 3.2 Den characteristics and surroundings

The choice of a den and the landscape features of its surroundings may affect individual fitness (Pigeon, Côté, & Stenhouse, 2016; Pigeon, Nielsen, Stenhouse, & Côté, 2014; Smereka et al., 2017). For example, if a pregnant female is forced to change her den during the winter, due to human disturbance or poor thermal qualities of the den, this can result in the loss of her offspring (Linnell et al., 2000). Females seem to show greater fidelity to denning area than males, as the same male individual is able to choose different dens at distances up to four times greater than that of females, for example, 1.7 versus 7.8 km (Linnell et al., 2000), while dispersing subadults do not seem to show fidelity to denning area (Manchi & Swenson, 2005). There is little or no reuse of the same den over successive years (Ciarniello et al., 2005; Elfström & Swenson, 2009; Schoen et al., 1987), although natural cavities seem to be more reused than excavated dens because the latter have lower structural stability (Linnell et al., 2000).

#### 3.2.1 Den structure

**FIGURE 3:**
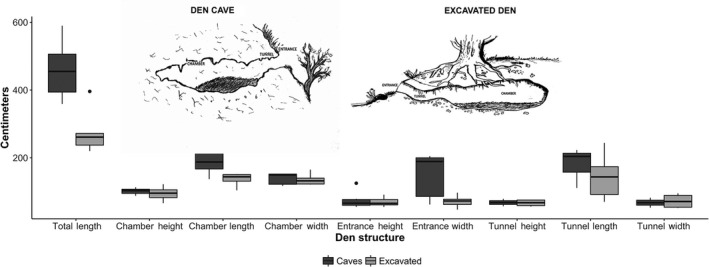
Dimensions (cm) of brown bear dens: excavated and natural caves. References for the Figure 3: (Chirichella et al., 2019; Clevenger & Purroy, 1988; Craighead & Craighead, 1972; Groff et al., 1998; Huber & Roth, 1997; Judd et al., 1986; Krofel et al., 2017; Lentfer et al., 1972; McLoughlin, Case, et al., 2002; McLoughlin, Cluff, et al., 2002; Miller, 1990; Petram et al., 2004; Pigeon, Côté, et al., 2016; Reynolds et al., 1976; Schoen et al., 1987; Vroom et al., 1980)

The most common den types are those excavated in the ground or located inside natural caves (Linnell et al., 2000). However, bears can also use other types of dens such as depressions under rock shelters, nest dens (a nest of needles and branches or other materials deposited on the ground), and tree cavities (Elfström & Swenson, 2009; Elfström, Swenson, & Ball, 2008; Seryodkin, Miquelle, Goodrich, Kostyria, & Petrunenko, 2018; Štofík & Saniga, 2012; Tammeleht, Kull, & Pärna, 2020). A common bear den is generally composed of three compartments (Figure 3): (1) an entrance; (2) a tunnel that connects the entrance with the resting chamber; and (3) a chamber occupied by the nest, that is, the zone where the bear gathers vegetative material to build a bed. Some structural parameters of dens, such as total length, tunnel length, chamber length, and width, may vary considerably (Figure 3), which might be due to the properties of the soil in excavated dens and brown bear adaptability when choosing natural cavities. The small size of the den, compared with that of the bear, allows greater thermal stability, and this is especially notable for excavated dens (Craighead & Craighead, 1972; Petram, Knauer, & Kaczensky, 2004). Some authors have suggested that although the construction of the den is an innate behavior programmed into the bear's genes, it may also be improved through experience acquired from the mother at the yearling stage (Craighead & Craighead, 1972; Petram et al., 2004; Vroom, Herrero, & Ogilvie, 1980). In addition, the features of the caves used by brown bears as dens do not seem the result of population‐specific traditions (Chirichella et al., 2019). Den construction can take place in only a few hours (Friebe et al., 2001), although bears can begin to prepare the den 1–2 months before den entry (Craighead & Craighead, 1972; Krechmar & Krechmar, 1992).

#### 3.2.2 Den landscapes

The choice of landscape surrounding the den is highly variable and mainly depends on the habitats available in the bear's home range (Ciarniello et al., 2005; Elfström et al., 2008; Linnell et al., 2000). Den selection is primarily the result of both broad‐ and fine‐scale habitat selection, mainly linked to den insulation, remoteness, and the availability of spring food resources at den emergence (Pigeon, Côté, et al., 2016; Pigeon et al., 2014). For example, the snowpack can have insulating properties that help maintain a constant temperature and in this way decrease the energy cost to the animal as a result of thermoregulation (Elfström et al., 2008; Lentfer, Hensel, Miller, Glenn, & Berns, 1972; Libal, Belant, Leopold, Wang, & Owen, 2011; McLoughlin, Case, et al., 2002; McLoughlin, Cluff, et al., 2002; Reynolds et al., 1976; Servheen & Klaver, 1983; Vroom et al., 1980). However, the role that snow might play when bears are inside the den is unclear. Thick forest cover may better protect dens from wind and cold temperatures (Pigeon, Côté, et al., 2016; Pigeon et al., 2014), and thick vegetation cover also guarantees concealment of the den entrance and, thus, protection from human disturbance (Chirichella et al., 2019; Sahlén et al., 2011). Finally, in the case of excavated dens, trees roots may help ensure greater structural stability of the den (Ciarniello et al., 2005; Harding, 1976; Judd et al., 1986; Lentfer et al., 1972; Smereka et al., 2017; Vroom et al., 1980). In the case of excavated dens, brown bears also choose hibernation areas on the basis of lithological characteristics, selecting soils that not only are easier to dig but also increase den stability and insulating capacity (García, Lastra, Marquínez, & Nores, 2007; Harding, 1976; Manchi & Swenson, 2005; McLoughlin, Case, et al., 2002; McLoughlin, Cluff, et al., 2002; Reynolds et al., 1976; Smereka et al., 2017; Vroom et al., 1980). Frequently, southeastern and southwestern slopes are preferred for greater insulation (Chirichella et al., 2019; Craighead & Craighead, 1972; Elfström et al., 2008; Harding, 1976; McLoughlin, Case, et al., 2002; McLoughlin, Cluff, et al., 2002; Schoen et al., 1987; Štofík & Saniga, 2012). Bears seem to prefer steeper slopes (Ciarniello et al., 2005; Elfström et al., 2008; Goldstein, Poe, Suring, Nielson, & McDonald, 2010; Libal et al., 2012; McLoughlin, Case, et al., 2002; McLoughlin, Cluff, et al., 2002; Pigeon et al., 2014; Servheen & Klaver, 1983; Smereka et al., 2017; Sorum, Joly, Wells, Cameron, & Hilderbrand, 2019; Štofík & Saniga, 2012; Vroom et al., 1980) which may allow for the following: (a) simpler digging of the den compared with flat ground (McLoughlin, Case, et al., 2002; McLoughlin, Cluff, et al., 2002); (b) greater den structural resistance, reducing the likelihood of collapse (Ciarniello et al., 2005; Libal et al., 2012; Servheen & Klaver, 1983; Vroom et al., 1980); (c) large amounts of radiant heat (Ciarniello et al., 2005; Libal et al., 2012; Vroom et al., 1980); and (d) better protection against disturbances (Libal et al., 2011, 2012; Naves & Palomero, 1993).

Den altitude varies from a minimum average height of 434 m a. s. l. to a maximum average height of 2,696 m (*n* = 22 studies). However, brown bears seem to avoid denning in valley bottoms and high peaks (Linnell et al., 2000). Bears might also select dens at higher elevations given that: (a) greater altitude allows dens to be covered by more snow and, consequently, to have greater thermal insulation (Eriksen, Wabakken, Maartmann, & Zimmermann, 2018; Libal et al., 2011, 2012; Sorum et al., 2019; Vroom et al., 1980; Whiteman et al., 2017), in addition to having fewer melting‐freezing events and better drainage (Eriksen et al., 2018; Whiteman et al., 2017); (b) dens at higher altitudes are further from the sources of human disturbance and are more inaccessible (Chirichella et al., 2019; Ciarniello et al., 2005; Pigeon et al., 2014; Whiteman et al., 2017); and (c) there are less natural predators, such as tigers (Seryodkin et al., 2003, 2018), at higher altitudes.

#### 3.2.3 Den selection and human disturbance

Humans may influence brown bear den selection (Craighead & Craighead, 1972). Actually, human activities and infrastructures may determine denning locations, for example, by increasing the distance from humans (Elfström & Swenson, 2009; Elfström et al., 2008; Eriksen et al., 2018) and/or by forcing bear to select concealed or inaccessible places. For example, winter dens close to human settlements or infrastructures (mainly roads) are typically located on steep (Eriksen et al., 2018; Groff, Caliari, Dorigatti, & Gozzi, 1998; Petram et al., 2004) and rugged (Eriksen et al., 2018; Sahlén et al., 2011) slope, as well as in areas with dense forest cover (Eriksen et al., 2018; Pigeon et al., 2014; Sahlén et al., 2011; Tammeleht et al., 2020). It has also been suggested that in places with a long history of persecution, such as Scandinavia, bears would select denning locations that are inaccessible or hidden (Eriksen et al., 2018).

#### 3.2.4 Den selection by class, age, and sex

Den selection may also vary according to the different classes of bears, as well as age and sex. As a general pattern, females seem to prefer dens at higher altitudes and with steeper slopes (Libal et al., 2011; Schoen et al., 1987), and this is particularly true for pregnant females, probably because these areas allow for a longer period of denning as a result of favorable thermal conditions, that is, longer snow cover at higher altitudes increases thermal insulation and reduces energy loss (Haroldson et al., 2002). In contrast, males may select areas at lower altitudes because of the greater availability of food at den emergence, which allows bears to rapidly increase body mass and thus improve breeding success (Libal et al., 2011). Yet, (1) females may also select areas within the tree‐line (Gardner, Pamperin, & Benson, 2014), (2) there are no differences between age classes in the selection of elevation and slope (Elfström & Swenson, 2009; Podruzny, Cherry, Schwartz, & Landenburger, 2002), and (3) males can hibernate in areas at higher altitudes than females (Seryodkin et al., 2003). Thus, whereas proximate factors might be at the origin of den selection for most brown bear classes, ages, and both sexes, pregnant females may select dens mainly on the basis of reproduction needs.

### 3.3 Hibernation physiology and the potential energetic costs

Brown bears hibernate for several months during which they do not eat, drink, defecate or urinate, thus reducing the use of the bladder, kidneys, and digestive tract (Folk, Folk, & Minor, 1972; Hissa, 1997; Stenvinkel, Fröbert, et al., 2013). Hibernation adaptations allow bears to overcome anuria, hyperlipidemia, and immobilization (Welinder et al., 2016), preserve muscles and bones avoiding osteoporosis or sarcopenia (Fröbert, Frøbert, Kindberg, Arnemo, & Overgaard, 2020; Vestergaard et al., 2011) and prevent diseases such as metabolic syndrome, diabetes, and cardiovascular pathologies (Arinell et al., 2012; Fröbert et al., 2020; Nelson & Robbins, 2015). The physiological modifications that occur in bears during hibernation, and which are summarized below, might be the result of ultimate factors such as metabolic dietary‐related needs and energy conservation, which brown bears have to deal with to survive the winter (Table 1).

**TABLE 1:** Brown bear hematology and coded references that show increases, decreases or nonsignificant differences of different compounds

Haematological compounds	Decrease during hibernation	Increase during hibernation	No significant variation between active period and hibernation
Glucose		7	
Fructose	7		
Urea	2, 1, 5, 4, 7		
Creatinine		2, 7, 10	4
Urea/Creatinine ratio	2, 4, 7		
Uric acid	7		4
Lactate		8	
β‐hydroxybutyrate		2, 10	
Potassium			10
Phosphorus	7		
Calcium		5	7, 10
Magnesium		10	
Vit. D2 and metabolites		6	
Vit. D3 and metabolites	5, 6		
D3/D2 ratio	6		
Osteocalcin	6		
Thyroxine (T4)	2		
Triiodothyroxine (T3)	2		
Thyrotropin (TSH)			2
β‐endorphins	2		
Parathyroid hormone (PTH)			5, 6
Aldosterone		5	
Total plasma protein		11	
Albumin		7, 10, 11	
Haptoglobin		3	
Total amino acids			7
Essential amino acids			7
Non‐essential amino acids			7
Branched chain amino acids			7
Cystine		2, 5	
Cysteine	9		
Lysine		5, 7	
Alanine		5	
Methionine	7, 13	2, 5	
Taurine	5, 7		
Arginine	5		
Asparagine	5, 7		
Leucine			5
Isoleucine			5
Threonine	7		5
Valine			5
Histidine		5, 7	
Phenylalanine		5	
3‐metylhistidine		2, 4, 7	
Ornithine		5	
Tryptophan			5
Glycine			5
Glutamine		7	
Glutamic acid		7	
Lipase			10
Kinase			10
Amylase	10		
Lactate dehydrogenase	10, 12		
Alanine transaminase	10		
Aspartate deaminase	10		
Alkaline phosphatase	10		
γ‐glutamyl transpeptidase	10		
Glutamate dehydrogenase	10		

Note

References for the Table 1: 1. Halloran and Pearson (1972); 2. Hissa et al. (1994); 3. Mominoki et al. (1996); 4. Barboza et al. (1997); 5. Hissa et al. (1998); 6. Vestergaard et al. (2011); 7. Stenvinkel, Fröbert, et al. (2013)); 8. Revsbech et al. (2013); 9. Revsbech et al. (2014); 10. Græsli et al. (2015); 11. Welinder et al. (2016); 12. Sommer et al. (2016); 13. Stenvinkel et al. (2018).

Although hibernating ursids share some adaptations with some smaller hibernators (e.g., squirrels, marmots, hamsters, and hedgehogs), such as storage and depletion of body reserves, lower metabolic rate, and body temperature during hibernation, many of the physiological changes of hibernating ursids are unique (Hellgren, 1998; Nelson & Robbins, 2015). For example, hibernating bears only experienced slight drop in body temperature (Hellgren, 1998), compared withthe greater body temperature drop of small hibernators (Carey, Andrews, & Martin, 2003; Nelson & Robbins, 2015). The metabolic rate drops in all hibernating mammals, but the mechanisms of metabolic rate reduction have been suggested to be different in bears (Evans et al., 2016; Geiser, 2004). Bears and small hibernators reduce cardiac output during hibernation from the active state, but the decrease is much greater in the latter (Nelson & Robbins, 2015). Actually, during the hibernation bears remain in an alert state and are thus able to rapidly increase their heart rate and mobility (Evans et al., 2012, 2016; Hissa et al., 1994), against the nonresponsive hibernating state exhibited by small hibernators (Nelson & Robbins, 2015).

The hibernating induction trigger (HIT), a compound present in the blood, might initiate physiological and metabolic changes that lead to hibernation (Hellgren, 1998; Hissa et al., 1994; Jørgensen et al., 2014; Welinder et al., 2016), although it has also been suggested than more than a single substance could trigger all these changes (Hissa, 1997). For example, the sex hormone‐binding globulin protein, which increases its concentration 45‐fold during hibernation, might also help trigger hibernation (Welinder et al., 2016).

#### 3.3.1 Energy consumption and changes in body mass and temperature

Hibernation requires a reduction of metabolic mechanisms with the consequent decrease in body temperature and consumption of O_2_. In hibernating bears, bradycardia results in a reduced volume of circulating blood and a lower rate of O_2_ consumption (Folk et al., 1972), and the metabolic rate falls to approximately 27% of basal rates (captive brown bears, Farley & Robbins, 1995). The metabolic cycle of brown bears, during which body mass variation occurs, can be divided into three stages: (a) gain of lean mass during spring, (b) accumulation of fat during hyperphagia, and (c) weight loss during hibernation (Hilderbrand, Jenkins, Schwartz, Hanley, & Robbins, 1999; Nelson et al., 1983). Evans et al. (2012) reported an increase in body mass of around 40% during the prehibernation hyperphagia phase for brown bears in Scandinavia. The rate of weight gain, which can reach up to 4 kg/day (Hilderbrand et al., 1999), depends on the size of the individual and their diet. During hibernation, losses of body mass can vary among latitudes (Swenson, Adamič, Huber, & Stokke, 2007) and between sexes (Kingsley, Nagy, & Russell, 1983: 18% in males vs. 40% in females), these losses being higher in pregnant females due to childbirth and cubs rearing (Hilderbrand, Schwartz, Robbins, & Hanley, 2000; Keay, Robbins, & Farley, 2018). Similar patterns were reported by Swenson et al. (2007), who compared winter weight losses between Scandinavian and Dinaric bear populations: weight loss varied between sexes and latitudes (26% males vs. 40% females in Scandinavia; 18% in both males and females in the Dinaric Mountains). The latitudinal variation has been suggested to be due to prolonged hibernation (Swenson et al., 2007), while the variations between sexes might be due to the frequency and duration of hibernation interruptions by males when food is available during winter (Krofel et al., 2017; Van Daele et al., 1990). In addition, differences across populations have been reported in the proportional body fat content and proportion of calories from fat, indicating certain plasticity according to seasonal food availability, reproduction, and climate (Hilderbrand et al., 2018). The decrease in body temperature during hibernation ranges from 3 to 5°C with respect to the body temperature of the active state (Hissa, 1997) and begins on average 13 days before den entry, while its recovery begins 63 days before den emergency (61.4% of the average hibernation time already completed, South‐Central Sweden; Evans et al., 2016).

#### 3.3.2 Lipid metabolism

The accumulated fat prior to hibernation plays three roles: (a) to supply the energy needs during hibernation, (b) to insulate the body of the bear helping to keep its temperature stable, and (c) to provide energy immediately after hibernation (Folk et al., 1972). Because hibernating brown bears maintain a fat‐based metabolism for several months, they exhibit an increase in plasma lipids (Arinell et al., 2012). High concentrations of phospholipids and cholesterol may be due to the shrinking of the membrane of the adipocytes caused by dehydration (Welinder et al., 2016). The high concentrations of free fatty acids (FA) and glycerol might be the result of their release from adipose tissue during hibernation (Welinder et al., 2016), with short chain (easier oxidation) of FAs being released and retained in muscle and tissue adipose than long FA chains, as it occurs in other hibernating mammals (Giroud et al., 2019). Long chain of FAs, such as Omega 3 and Omega 6, some of which are responsible for carbohydrate metabolism and protein sparing in bear muscles (Chazarin, Storey, et al., 2019), vary their concentration in muscles and plasma differently between the active and hibernating states (Giroud et al., 2018). However, metabolites as eicosanoids decrease or do not vary their concentration during hibernation (regardless of their pro or anti‐inflammatory properties), suggesting that the hibernation period is associated with a depressed state of the eicosanoid cascade (Giroud et al., 2018). Adiponectin is secreted exclusively in adipose tissue and is responsible for inducing insulin resistance to regulate the oxidation of fatty acids (Havel, 2002; You, Considine, Leone, Kelly, & Crabb, 2005), which may help maintain lipogenesis during the hyperphagia when bears need to store fat, whereas the decrease during hibernation that generates insulin resistance facilitates the switch to a lipolytic metabolism (Kadowaki et al., 2006). Leptin has a regulating function of appetite, so that increased serum concentration decreases food intake (Trayhurn, Hoggard, Mercer, & Rayner, 1999; Wang, Walter, Bhat, Florant, & Coleman, 1997). Some studies have described the temporary insensitivity of brown bears to leptin during hyperphagia, with a peak at the end of this period and, consequently, a sharp decrease in appetite at the moment of den entry (Nelson et al., 1983).

#### 3.3.3 Metabolism of nitrogenous substances and turnover of protein compounds

During hibernation, when bears do not excrete waste in the form of urine or excrement (Stenvinkel, Jani, & Johnson, 2013), their bladder becomes permeable and both water and nitrogenous substances of the urine (such as urea) re‐enter the blood (Brown, Mulhausen, Andrew, & Seal, 1971; Nelson, Jones, Wahner, McGill, & Code, 1975). Therefore, bear physiological adaptations allow recycling of nitrogenous substances, such as urea or creatinine, preventing the development of renal complications or azotemia (Barboza, Farley, & Robbins, 1997; Brown et al., 1971; Stenvinkel, Jani, et al., 2013; Stenvinkel et al., 2018).

The urea content of blood plasma decreases during the hibernation period (Brown et al., 1971; Halloran & Pearson, 1972; Hissa, Puukka, Hohtola, Sassi, & Risteli, 1998; Hissa et al., 1994), being up to two times less than that outside hibernation (Stenvinkel, Fröbert, et al., 2013). It has been observed that urea decrease begins in autumn, probably due to a higher intake of fruits and berries, which are poor in protein (Welch, Keay, Kendall, & Robbins, 1997). The mechanism by which hibernating bears reduce their urea levels is unique (Nelson et al., 1975; Stenvinkel, Jani, et al., 2013). It is based on a reduction of urea synthesis in the liver and its recycling by reincorporating urea into skeletal muscle and other proteins (Nelson et al., 1975; Stenvinkel et al., 2018), and the possible conversion of part of the urea into ammonia and CO_2_ by gut microbiota (Barboza et al., 1997; Hellgren, 1998). This mechanism would have the added advantage of preventing the loss of muscle tissue during hibernation (Stenvinkel et al., 2018). Parallel to the decrease in urea levels, there is an increase in creatinine (Græsli et al., 2015; Hissa et al., 1994), which can more than double, as it cannot be eliminated given that it is not metabolized during hibernation and there is no excretion (Stenvinkel, Fröbert, et al., 2013). Thus, there is a change in the urea/creatinine ratio from the active period to hibernation (Hissa et al., 1994; Stenvinkel, Fröbert, et al., 2013). The synthesis of urea in the liver decreases as part of metabolic energy saving during hibernation (Stenvinkel, Fröbert, et al., 2013) and as a result of the change from a carbohydrate and protein metabolism to a lipid metabolism (Græsli et al., 2015).

Different studies have reported very different trends regarding the seasonal variations of plasma proteins and amino acids (Table 1). Despite not consuming any protein during the fasting that accompanies hibernation, the protein content of serum decreases little during this period (4%–17%; Chanon et al., 2018) and increases in the case of some proteins and amino acids (Table 1). Hellgren (1998) even suggested that increases in protein metabolism could (a) prevent its catabolism into carbon dioxide, water, and urea and (b) supply the needs of specific enzymes such as lipolytics, gluconeogenic, or proteolytic.

#### 3.3.4 Response of the circulatory system to hibernation

A multitude of cardiovascular and hematological adaptations of the circulatory system occur during hibernation, many of them aimed at conserving energy (Jørgensen et al., 2014). Heart rate begins to decrease 24 days before den entry, whereas recovery of cardiac parameters starts 33 days before den exit (unanesthetized bears, South‐Central Sweden Evans et al., 2016). However, despite the fact that brown bears (a) exhibit blood parameters that would be indicators of pathology in humans and (b) maintain levels of cholesterol and triglycerides much higher than healthy human values, atherosclerosis, fatty streaks, foam cell infiltration, and inflammation have not been reported, and coronary artery examination has revealed the absence of atherosclerotic changes (Arinell et al., 2012). Deep bradycardia leads to a reduction in blood flow, which decreases the low shear stress in blood vessels, a factor that is related to atherosclerotic plaque (Jørgensen et al., 2014).

As a result of a decrease in metabolic rate and activity, hibernating brown bears exhibit marked bradycardia in which the heart rate decreases between 63% and 80% (unanesthetized bears: (Evans et al., 2016); anesthetized bears: (Folk et al., 1972; Jørgensen et al., 2014, 2020; Nelson & Robbins, 2010)). However, blood pressure does not seem to change during hibernation (Nelson, McEwen, Robbins, Felicetti, & Christensen, 2003). Cardiac output has been reported as significantly lower during hibernation, representing only 24% of the active period value (0.86 vs. 3.54 L/min). Another measure of cardiac activity, the cardiac index, is also lower in brown bears during hibernation, with a value nearly only a quarter (26%) of that for the active period (2.45 vs. 0.63 L min^−1^ m^−2^). Stroke volume also varies, decreasing ca. 69% during hibernation, which is consistent with adaptation to low energy demands (Jørgensen et al., 2014).

#### 3.3.5 Skeletal response to hibernation, bone turnover and changes in skeletal muscles

The periods of shivering experienced periodically by hibernating bears, which help maintain muscle function, may also generate skeletal loading on bone to preserve its properties (Lin, Egeland, Schertenleib, Nelson, & Robbins, 2004). The concentration of the parathyroid hormone, which stimulates osteoclast activity and weakens bone by releasing Ca^2+^ into the blood, does not vary significantly between summer and winter (Table 1), consistent with the similar levels of Ca^2+^ in the blood between seasons (Græsli et al., 2015; Hissa et al., 1998; Stenvinkel, Fröbert, et al., 2013; Vestergaard et al., 2011). On the other hand, some authors described a two‐fold lower concentration of osteocalcin (the hormone indicative of bone formation; Vestergaard et al., 2011) and a decline of alkaline phosphatase (the hormone related to bone formation) during hibernation (Græsli et al., 2015). Brown bear bones do not lose their mechanical function despite inactivity (McGee‐Lawrence, Carey, & Donahue, 2008). An increase in trabecular remodeling (the trabecular bone is formed of interstitial septa called trabeculae, forming a spongy structure) might allow for the maintenance of trabecular structure and Ca^2+^ homeostasis, since bears cannot excrete the latter during hibernation (Floyd, Nelson, & Wynne, 1990), and it is considered the main factor in maintaining bone health (Stenvinkel et al., 2018).

Brown bears show no noticeable loss of muscle function or marked atrophy during hibernation (Salmov et al., 2015). During hibernation brown bears experience shivering (McGee et al., 2008), with periods that can exceed an hour in duration where activations lasting less than 0.2 s and occur every 3–10 s. Shivering may stimulate skeletal muscles enough to maintain muscular fitness (captive brown bears, Lin, Hershey, Mattoon, & Robbins, 2012). It has also been suggested that the plasma of hibernating bears has antiproteolytic properties, thus inhibiting muscle loss (Chanon et al., 2018; Fuster, Busquets, Almendro, López‐Soriano, & Argilés, 2007; Salmov et al., 2015) and that constant levels of prostaglandins in muscle could contribute to muscle sparing in bears (Giroud et al., 2018). Skeletal muscles in hibernating brown bears are alleviated from oxidative stress, through the increased expression of cold‐inducible proteins, and from a reduced production of reactive oxygen species (due to metabolic suppression and increased activity of antioxidant systems), which would confer resistance to skeletal muscle atrophy (Chazarin, Ziemianin, et al., 2019). The maintenance of glycolysis would contribute maintaining functionality in cases of rapid den exit and fast increase in ATP production (Chazarin, Storey, et al., 2019). Finally, higher levels of metabolic microRNAs during hibernation have been reported to be responsible for metabolic suppression and for the activation of myogenic pathways, decreasing atrophic signaling (Luu et al., 2020). Taken together, these findings suggest that brown bears are able to maintain both muscle mass and function by reducing catabolic processes and maintaining a certain level of mechanical activity.

#### 3.3.6 Liver and kidney changes during hibernation

Before fasting, anuria and decreased metabolic rate, organs involved in digestive, metabolic and excretion processes, such as the liver and kidneys, have their activity modified compared with the active period (Græsli et al., 2015; Stenvinkel, Jani, et al., 2013). Although the maintenance of circulating urea is indicative of a functioning liver during the hibernation of bears (Barboza et al., 1997), decreases in the levels of alkaline phosphatase (Græsli et al., 2015; Table 1) and the concentration of bile acids occur (Lin et al., 2012), consistent with fasting and hypometabolism (Græsli et al., 2015; Sommer et al., 2016), a condition that has also been suggested for the pancreas due to the decrease in amylase concentration in serum (Græsli et al., 2015).

Perls‐positive granules, indicative of the accumulation of stainable ferric iron, appear in the cytoplasm of Kupffer cells, as well as in other nonparenchymal cells and some hepatocytes, and central veins are partially pleated at den emergence, with the narrowest lumen diameters and portal veins partially fibrosed (Prunescu, Serban Parau, Prunescu, Brock, & Vaughan, 2003). This increase in Fe may be due to the inability of excreting it and weight loss, as Fe is phagocytized by the Kupffer cells without recycling to other organs, whereas the narrowing of the hepatic vessels may be due to the smaller volume of blood during hibernation, which may not be sufficient to preserve normal form and thus results in pleating.

The kidney has a reticulated structure with separate lobes that decrease resistance to intraluminal flow. A decrease of 90% in renal flow has been reported, indicating a decrease in function, due to the lack of water intake and a decrease of 50%–70% in glomerular filtration rate (Stenvinkel, Fröbert, et al., 2013; Stenvinkel et al., 2018). Concentrations of urea and creatinine in the blood have been suggested as good indicators of renal function, and high values of these compounds could reflect impaired renal function (Stockham & Scott, 2008). On the one hand, the very high concentrations of creatinine and magnesium in blood plasma (Table 1; Græsli et al., 2015; Hissa et al., 1994; Stenvinkel, Fröbert, et al., 2013) have also been suggested as indicators of decreased function of the kidneys, since creatinine and magnesium are filtered from the blood by these organs (Græsli et al., 2015). On the other hand, although the glomerular filtration rate is reduced and the kidneys do not excrete during the hibernation process, serum urea decreases, indicating a unique ability of brown bears to recycle urea in protein compounds (Nelson, Wahner, Jones, Ellefson, & Zollman, 1973).

#### 3.3.7 Pregnancy and lactation

Due to the difficulty of gaining access to free‐ranging pregnant females and the invasive nature of their study in natural conditions, very few studies have been published on pregnancy and lactation in free‐ranging bears. Therefore, the information that exists about these processes during hibernation in free‐ranging brown bears is very scarce.

Brown bear females may go into heat from late spring to late summer (Fernández‐Gil et al., 2006; LeFranc, Moss, Patnode, & Sugg, 1987) and give birth during hibernation in mid‐winter, around January–February, during a period when there is no intake of food and water (Farley & Robbins, 1995). Although it has been suggested that the implantation of the blastocyst is delayed until den entry (Hensel, Troyer, & Erickson, 1969), in one study conducted on free‐ranging parturient females in Sweden, Friebe et al. (2014) found no correlation between the date of delivery and the den entry, suggesting the influence of factors other than the start of denning on blastocyst implantation. These authors report December 1 as the average implementation date and January 26 as the average date of parturition, with an average gestation duration of 56 days. This same study reports that body temperatures of pregnant females are higher during the gestation period than during the rest of hibernation, to then drop at parturition due to fetal development. In older females, as well as in females with high body fat content, (a) den entry dates and birth dates tend to be earlier than in other females, (b) the lactation period may be longer (Friebe et al., 2014), and (c) implant embryos and cub birth occur earlier. Females with high body fat content also produce more and better milk than lean mothers (Hissa, 1997; López‐Alfaro, Robbins, Zedrosser, & Nielsen, 2013; Robbins, Ben‐David, Fortin, & Nelson, 2012). Finally, the mortality of cubs during the first summer is lower in females with a higher percentage of fat and lean mass (Keay et al., 2018).

## 4 DISCUSSION AND CONCLUSIONS

We suggest that ultimate factors, such as (a) circannual changes in climate, (b) metabolic dietary‐related needs, (c) energy conservation (necessary for increasing the probability of survival despite limited food availability in winter), and (d) female pregnancy, over an evolutionary timescale, have shaped physiological mechanisms that make hibernation beneficial to brown bears. On the other hand, the main proximate factors of hibernation, which include current weather conditions and food availability, also contribute to triggering physiological mechanisms that initiate hibernation. Yet, there are several physiological correlates of hibernation that, along with the environmental conditions that trigger hibernation, may be considered proximate causes. Because proximate mechanisms that regulate hibernation are superimposed upon regulated circannual changes in appetite, body mass, reproduction needs and several physiological processes, it is important to correctly distinguish between actual drivers of hibernation, that is, factors to which bears directly respond (temperature, snow, food availability) and their correlates, that is, factors that may be correlated to actual drivers (physiological changes; (Carey et al., 2003)). However, since hibernation can be a flexible response, we suspect that the correlates of proximate factors might fluctuate according to current environmental variations.

Predicted variations in air temperatures generally point toward an increase in temperatures (IPCC, 2013; Raftery, Zimmer, Frierson, Startz, & Liu, 2017), with a predicted increase of 2–4.9°C in global average temperature by 2,100 (IPCC, 2013). For example, the rise of mean temperatures, in addition to the increase in temperature and precipitation variability (Giorgi, Bi, & Pal, 2004; Pendergrass, Knutti, Lehner, Deser, & Sanderson, 2017), has already affected biological systems by altering the phenology of seasonal processes (Root, Price, Hall, & Schneider, 2003). Interannual fluctuations in hibernation chronology are expected to occur due to interannual variations in climate, extreme climatic events, and temperature anomalies resulting from climate change (Giorgi et al., 2004; Pendergrass et al., 2017). Moreover, the increased climatic variability could make the weather patterns that govern the seasonality of animal life cycles to some extent more unpredictable for many organisms, including bears (Weiskopf et al., 2020). Actually, these interannual fluctuations in hibernation chronology due to climatic conditions have already been reported in both American black bears (Miller, Smith, Auger, Black, & Allphin, 2017) and brown bears (Delgado et al., 2018; Evans et al., 2016; Friebe et al., 2014; McLoughlin, Case, et al., 2002; McLoughlin, Cluff, et al., 2002; Pigeon, Stenhouse, et al., 2016). Pigeon, Stenhouse, et al. (2016) reported that for each 4°C increase in spring temperature brown bear den exit occurs 10 days earlier. Thus, changes in climate could reduce the duration of hibernation in bears and lead to advanced den exit (Berman, Coops, Kearney, & Stenhouse, 2019; Johnson et al., 2017; Pigeon, Stenhouse, et al., 2016). It has been reported how brown bears emerge from winter dens when the ambient temperature reaches a certain level (3.7 ± 1.3 C in Evans et al., 2016), and since warmer springs may promote earlier first den exits (e.g., Delgado et al., 2018; González‐Bernardo et al., in press), bears are expected to emerge from dens earlier as the climate continues to warm (Johnson et al., 2017; Pigeon, Stenhouse, et al., 2016). Although few data are available, it has been suggested that: (a) climate change may severely reduce the available spring food resources (Holden, Kasworm, Servheen, Hahn, & Dobrowski, 2012; Inouye, Barr, Armitage, & Inouye, 2000; Penteriani, Zarzo‐Arias, Novo‐Fernández, Bombieri, & López‐Sánchez, 2019; Roberts, Nielsen, & Stenhouse, 2014) or produce a temporary change in its availability (Deacy et al., 2017); and/or (b) if bears exit dens earlier, vegetation production may still not be sufficient to support their food requirements. This may be more important for populations that exhibit a lower altitudinal difference between denning and spring foraging habitat. Moreover, such increased mismatches might increase the likelihood of bear‐human conflicts if bears emerge earlier and, thus, have fewer foraging options over a more protracted time. An increase in winter temperatures would have a negative effect on reproductive success and cub survival after den exit in brown bear populations: energy demands of hibernating mammals would increase with higher winter temperature, due to the increase of energetic costs of torpor (Albrecht et al., 2017; Humphries, Thomas, & Speakman, 2002; Post & Forchhammer, 2008; Turbill & Prior, 2016). Moreover, it has been suggested that an early den exit might also have negative consequences on the physical condition of cubs at den emergence, and therefore their fitness, as cubs that leave prematurely may be smaller and thus more vulnerable to predation or infanticide (Bellemain, Swenson, & Taberlet, 2006; Hertel et al., 2018; Pigeon, Stenhouse, et al., 2016). Thus, it is crucial to understand how changes in climatic factors might affect the ability of bears to cope with global climate change. Yet, understanding the relationship between hibernation and global warming is essential for brown bear conservation and management in a changing world as climate‐induced changes in hibernation have the potential to affect individual and population fitness (Delgado et al., 2018; Hertel et al., 2018; Pigeon, Stenhouse, et al., 2016).

Brown bears have been reported showing a noticeable plasticity when hibernating, adapting their denning behavior to environmental factors, availability of food during hyperphagia or changing snow conditions during the winter (Fowler, Belant, Wang, & Leopold, 2019). However, it is unknown how adaptable brown bears can be to changes in food availability or climate regimes (Hertel et al., 2018). When this variability is predictable, as in populations where supplementary feeding is provided, populations have demonstrated a rapid adaptation of the hibernation chronology (Bojarska et al., 2019; Krofel et al., 2017). If these changes are less predictable, we lack information on how bears might adapt to these year‐to‐year fluctuations. Because (a) early den exit by females with cubs may have repercussions on the health of cubs (Bellemain et al., 2006; Hertel et al., 2018; Pigeon, Stenhouse, et al., 2016) and (b) den abandonment of pregnant females increases probability of cub mortality (Swenson et al., 1997), (1) the autumn hunting season should end early enough so as to avoid disturbing female bears that have already denned or are showing predenning behavior (Friebe et al., 2001; Lodberg‐Holm et al., 2019), and (2) winter‐early spring human activities should be minimized near suitable or traditional denning sites (Linnell et al., 2000).

It is important to highlight here the potential effect of anthropogenic food, and especially supplementary feeding, on the chronology of hibernation. Supplementary feeding of bears has several purposes including hunting, eco‐tourism, and the mitigation of human–bear conflicts (Penteriani et al., 2017, 2018). In areas where brown bears have access to anthropogenic food, shorter denning periods (over 50% reduction in denning period) or greater winter den abandonments than in populations located at a similar latitude where these food sources do not exist have been reported (Bojarska et al., 2019; Krofel et al., 2017; Špacapan, 2012). Since supplementary feeding encourages bears to be active at an unusual time of year, one would also expect an increase in conflicts at a time of year when they are absent or less frequent. A decrease in the length of the hibernation period might also have unexpected and overlooked effects on bear physiology and behavior. Thus, more research is needed on the possible effects of supplemental feeding on denning behavior.

Little information exists on the predenning stage, which is also important because individuals start to approach denning sites and seem to alter their movement patterns and rhythms of activity (Friebe et al., 2001; Manchi & Swenson, 2005; Sahlén et al., 2011). More research is needed on this phase, which represents the link between hyperphagia and the moment of den entry.

In studies on denning, we risk focusing on correlated factors erroneously assuming they are true drivers, while we ignore the actual drivers. Clearly, it may prove to be difficult to distinguish between a true driver and its proxies, but insights from physiology might help in this regard, providing the opportunity for a unifying approach that merges insights from different perspectives and disciplines, that is, physiology, ecology, behavior. Also, it is very difficult to do these studies in the field; we rely on biologging and environmental data, often not even at the bear's den. As bears dens are not selected prior to denning, and the brown bear is extremely sensitive to disturbance, few researchers have data on environmental conditions at the den site (or in the den).

Finally, to our knowledge, almost no information exists on the hyperphagia, predenning and denning periods for those (southernmost and coastal) populations where hibernation does not occur every year and/or is only performed by part of the population or just some bear classes, for example, pregnant females (but see Fuchs et al., 2019; Huber & Roth, 1997; Krofel et al., 2017; Nores et al., 2010; Van Daele et al., 1990). Although brown bears are not obligate hibernators, hyperphagia, and predenning are expected to prepare individuals to spend a considerable portion of their annual cycle in dens. Thus, information on movement patterns, rhythms of activity, and the physiology of individuals that do not hibernate (or hibernate for short periods) may allow for useful comparisons with those brown bears that may spend up to six‐month hibernating.

## CONFLICT OF INTEREST

None declared.

## AUTHOR CONTRIBUTION

**Enrique González‐Bernardo:** Conceptualization (supporting); Data curation (equal); Formal analysis (supporting); Investigation (equal); Writing‐original draft (lead); Writing‐review & editing (lead). **Luca Francesco Russo:** Data curation (equal); Formal analysis (supporting); Investigation (supporting); Writing‐original draft (equal); Writing‐review & editing (supporting). **Esther Valderrábano:** Writing‐original draft (supporting); Writing‐review & editing (supporting). **Ángel Fernández:** Writing‐original draft (supporting); Writing‐review & editing (supporting). **Vincenzo Penteriani:** Conceptualization (lead); Data curation (lead); Formal analysis (lead); Funding acquisition (lead); Methodology (lead); Project administration (lead); Resources (lead); Supervision (lead); Validation (lead); Writing‐original draft (equal); Writing‐review & editing (lead).

## Data Availability

The bibliography consulted in this review has been obtained through Scopus and accessed from the main repositories.
